# Consumption of legumes in children from 3 to 6 years – evaluation of an intervention program

**DOI:** 10.1093/eurpub/ckac129.514

**Published:** 2022-10-25

**Authors:** A Amaral, A Carvalho, J Lima

**Affiliations:** 1 Polytechnic Institute of Coimbra, ESTeSC - Coimbra Health School, Coimbra, Portugal; 2 GreenUPorto, Sustainable Agrifood Production, Porto, Portugal; 3 ciTechCare, Center for Innovative Care and Health Technology, Porto, Portugal; 4 Polytechnic Institute of Coimbra, Labinsaúde, Coimbra, Portugal; 5 HL, Leiria Hospital Center, Leiria, Portugal

## Abstract

**Introduction:**

Eating habits are a key aspect of a healthy lifestyle. This study focuses on the importance of consuming legumes - rich and accessible source of protein, and a healthy and sustainable option, in environmental terms - contributing to increment health literacy levels at the population level.

**Objectives:**

To design, implement and evaluate a program to promote the consumption of legumes - Beans4Life. Specifically, to assess its impact on the knowledge and frequency of consumption of the eight legumes (beans, grain, peas, beans, beans, lentils, chickpeas and lupins).

**Methods:**

Pre-test post-test analytical study, with three evaluation moments: 1) before the intervention; 2) after the intervention with the children and 3) after the intervention with the guardians (end of the intervention). Participants: 90 children from 3 to 6 years old (54.4% male) and their guardians. Instruments: questionnaire to assess knowledge and food preferences, questionnaire on eating habits and a questionnaire to evaluate sessions. The intervention had two components, the first with the children (4 food education sessions) and the second with the families (activities that facilitate the inclusion of recipes with legumes in the family's daily life).

**Results:**

Before the intervention, the results obtained show low levels of knowledge and frequency of consumption of legumes, influencing low health literacy. Peas and beans are the best known legumes (81.1% and 55.6%) and also preferred (77.8% and 73.3%). The results obtained in the second and third assessments show that there was a significant increase (p < 0.05) in the knowledge and frequency of consumption of most legumes.

**Conclusions:**

The objectives initially proposed were achieved and it will be pertinent to continue the present study, with larger samples, in order to increase health literacy awareness and the consumption of this food group in childhood, and provide more robust results that help to clarify the relationships found.

